# Career perspective: Alf O. Brubakk—looking back to see ahead

**DOI:** 10.1186/s13728-015-0023-z

**Published:** 2015-03-12

**Authors:** Alf O Brubakk

**Affiliations:** Department of Circulation and Medical Imaging, Faculty of Medicine, Norwegian University of Science and Technology, Trondheim, Norway; Comparative Medicine Lab, Institute of Clinical Medicine, University of Aarhus, Aarhus, Denmark

**Keywords:** Environmental physiology, Cardiology, Anaesthesiology, Diving

## Abstract

The following describes my professional life up till today, but it also describes what I think lies ahead. I have led an interesting professional life and been lucky enough to be at the centre of some of the important development in modern medicine and diving, namely ultrasound in cardiology and the mechanisms of decompression. I therefore should be able to see some of the most challenging and exciting problems ahead.

Ultrasound in cardiology has developed from simply listening to the Doppler signal to determine the velocity of blood flow to the complicated description of images presented today. Diving, in addition to being an important commercial and environmental activity, exposes the individual to intermittent hyperoxia and pressure reductions. These challenges evoke the production of radical oxygen species (ROS) and microparticles (MP) that also are central to many pathophysiologic mechanisms that are involved in a number of severe human diseases. Thus, diving can be regarded as an important model of disease and allows us to study their effects on healthy young individuals.

The future thus points towards an integration of environmental physiology with detailed physiological and pathophysiological mechanisms and makes diving physiology a potentially very important field of study.

## Career perspective

Medicine was not my first choice for a career. During the summer at my last year in high school, I worked as a research assistant on a research ship in the Barent’s sea; my job was to measure the size of fish caught in a trawl net. I was fascinated by the work here and wanted to be a marine biologist. However, I did not dare to go that way; I thought that if I was not good enough, I could only get a job as a teacher in high school! I wanted to avoid that, so I thought Medicine was a safer bet, since as a medical doctor you could always get an interesting job, I thought. My grades in high school were not good enough to study medicine in Norway so I went to Giessen in Germany.

In many ways, this was a lucky choice. Giessen was an old university town that had been bombed flat during World War II. It had just been re-opened when I came there. This meant that it had mostly young professors; the plans for study were constantly changing. Furthermore, Giessen was in Hessen that at that time was very progressive with students being elected to sit on various boards, including the University Council; I represented the students here for several years. I also was elected on the committee that was writing the study plans for the new faculty at the University of Ulm. As a medical student, I was also lucky enough to be able to go to Lindau to participate in the “Nobelprize Traeger Tagung”. Here, a large number of winners of the Nobel Prize in medicine met in a very informal atmosphere. I was struck by the different approaches to science that all led to top grade results. It also showed me the importance of freedom to follow your own road.

I was also very active in Association Norwegian Students Abroad (ANSA) where I ended up as Chairman of the Council and responsible for looking after medical students at a number of universities in Europe.

After graduating, I did my obligatory internship at a small island in West Norway. With no supervision, I had to find my own way. Hopefully I did some good and luckily I did not kill anybody.

At that time, there were only two medical faculties in Norway, Bergen and Oslo. I knew that more faculties would be established; I was particularly interested in the one in Trondheim, because this was the site of the Technical High School, NTH. My interest was in this direction, and I thought I could do my part to make a faculty of medicine here happen. I therefore initiated an ANSA conference in Trondheim that we called “Health care towards 2000”, where we managed to invite a significant number of leading politicians and educators.

At the end of this conference, I was approached by Sjur Børve, the head of the health service in Trondheim. He asked me if I would consider a research position financed by the county; my job was to try to get a medical faculty in Trondheim by establishing co-operation with NTH. At that time, I could not even spell research, but thought this would be a real challenge.

But again I was in luck. One of my friends told me that there is a mad student at NTH who claims he can make a mathematical model of the cardiovascular system. This is how I met Rune Aaslid.

What we wanted to do was to develop a circulatory computer model that could be used clinically. The ambitious idea was to perform measurements on the model that we could not perform in a patient then use the model to obtain the data that we could not measure in the patient.

We had no money and no credentials, but we had an idea; to many, we were considered crazy. I remember that one of the heads of the Norwegian Research Council came to us after we had applied for money that we did not get, “how do we know that you are not crazy??”. I had to admit we might be, and I could well understand that they did not give us any money.

Money came from many unlikely sources; the first was a contribution from the husband of a patient where we measured blood flow, another from the owner of the only shop in Trondheim who sold condoms.

However, we had also other supporters; one was Forenede Liv (an insurance company). We were also lucky enough to have the support of Professor Jens Glad Balchen at the Department of Cybernetics. He supported “mad” ideas and was a great believer in mathematical models, and he gave us working space at his department. Without his support, we would never have succeeded. That our idea was right has now been verified by a very significant and much more ambitious EU project called “The Digital Man”. So it shows the importance of not giving up and to believe in your ideas regardless of opposition.

In 1970, when our project was initiated, there was no digital computer that could solve the non-linear equations involved in our model. Rune built our own analogue computer that we named Jenny after the first patients that we investigated with the model.

Studying the model, it came clear to us that we needed more measurement data. We understood that ultrasound could be a very useful tool for obtaining data about the cardiovascular system. No equipment was commercially available, so we constructed our own equipment, pulsed echo Doppler flowmeter (PEDOF). A number of students were involved in this, among others were Kjell Kristoffersen, Bjørn Angelsen and Hans Torp. Using this equipment, we were able to record blood flow velocity in the aorta and the heart. One of the very useful outcomes of this work was that blood flow velocity could be used to evaluate pressure in the heart by using the Bernoulli equation. This method was developed by Jarle Holen and led to a work that has made ultrasound to obtain intra-cardiac pressure non-invasively possible, thus avoiding heart catheterisation [1].

Even if Jenny never was used in the way we envisaged, our insight here and the development of ultrasound have been a great success. Responsible for this early work was a number of very talented researchers; in particular, I will mention the work of Bjørn Angelsen and Liv Hatle. The work led to the establishment of a Norwegian Company Vingmed, now taken over by Generel Electric, GE. This is now one of the major producers of ultrasonic equipment for studying the heart.

After some years of research in cardiology, in particular related to the study of cardiovascular dynamics, I finished my doctoral thesis “Methods for studying flow dynamics in the left ventricle and the aorta in man; use of a simulation model and ultrasound, Trondheim 1978”

I then worked as an anesthesiologist for a number of years before I was offered a job as Assistant Professor of Applied Physiology in Trondheim.

I had a good friend who was an experienced diver, Bård Holand, who regretfully recently died. He suggested to me that my experience in ultrasound could be very useful for studying decompression in diving. At this time, Norway was at the beginning of oil exploration and production offshore; this made commercial diving an important activity. In Bergen, they had established an institute of diving research, Norwegian Underwater Institute, NUI. I joined them where we performed a number of experimental dives down to 500 msw. I performed a number of ultrasonic studies here. At this time, none of the so-called diving experts saw any value in looking for bubbles; “we have seen bubbles but they do not mean anything” was the message. I thought they were wrong, suggesting that bubbles had to have a pathophysiologic meaning.

I knew enough about diving to understand that decompression sickness, DCS, was a significant problem that was still not properly understood. I knew from my work with ultrasound that gas bubbles were a powerful reflector and that ultrasound could be used to detect bubbles. The first study showed that dives following accepted procedures led to significant bubble formation both on the arterial and venous side [2]. In spite of this, when the new decompression tables were developed by the US Navy, no consideration was taken of this. Only recently has the US Navy seen the value of considering vascular bubble formation [3].

This led me to a number of studies in decompression and bubble detection which are still ongoing. This has led to the development of Copernicus, a model that uses vascular bubbles as the endpoint of decompression.

For over 35 years until 2015, I have been the medical advisor to a major professional diving company ending with me being responsible in diving procedures worldwide for Subsea7. I have also run a number of diving courses.

We studied a number of various animal models of decompression. However, one of the most interesting developments came from my work by combining the bubble work with the studies of exercise physiology. The relationship between physical activity and decompression is still not completely clear; one of my friends, Ulrik Wisløff, had developed a model of exercise in rats, and we decided that it would be of interest to look at the effect of exercise in decompression. To our surprise, there was no significant effect. Looking closely at the data, we understood that the rats that had exercised on Friday and dived on Monday produced many bubbles, while those who had exercised the day before the dives had no bubbles. Furthermore, all animals that had rested over the weekend all died, while those which had exercised all survived. Further studies showed that the mechanism was related to the production of nitric oxide, NO; for the first time, it had been possible to show that decompression bubbles was dependent on biochemistry [4,5].

Many studies along these lines have been performed; the work of Steve Thom in particular has shown that decompression is an inflammatory disease. This has considerable potential as we have a considerable number of tools to study inflammation.

My own work along these lines has convinced me that hyperoxia and the production of radical oxygen species (ROS) is a central mechanism in the development of decompression problems.

The interest in diving has led me to study the effect of the environment in general. Every year for nearly 20 years, we ran courses on health and survival in the cold on Svalbard. We also got interested in the effect of hypoxia, leading to the hypothesis that pulmonary altitude sickness is caused by pressure reduction and thus is an altitude decompression sickness.

Over the years, I arranged a number of symposia in diving, resulting in several books. I was one of the editors of the 5th edition of The Physiology and Medicine of Diving.

The above focus on the belief that gas bubbles is the main problem in decompression. This has been the main theoretical basis for all decompression procedures since Haldane’s ground-breaking publication in 1908. A recent work indicates that this is not the whole story and may not even be the most important part of the story. A recent work indicates that the basis for the decompression problem is established already at pressurisation, with the breathing of hyperoxic gas. This starts a process, where bubble formation at decompression will have an effect depending on the state of the organism prior to decompression. This work focuses on the effects on the vascular system, where the effect on the endothelium is of importance. Based on this work, it can be stated that “DCS is an inflammatory disease”. If this hypothesis is correct, it will have wide ranging consequences, both for prevention of injury and treatment.

Diving is a relatively new development in the evolution of land living animals. One would have expected that mammals that live in the seas, like whales and seals, are adapted by evolution and thus do not form bubbles on decompression. We now know that this is not the case; bubbles are probably regularly formed, without harming them following normal diving. These animals do their dive on a single breath of air, carrying their oxygen store from the surface. Thus, these animals have established methods to prevent or reduce bubble effects [6]. We may presume that this ability is also present in man, perhaps in a more rudimentary form.

### Brief career summary

In my professional life, I have moved from cardiology and anesthesiology to environmental physiology and commercial diving. The changes in career were mostly governed by chance and opportunities. The most significant change was when I got involved in decompression problems and commercial diving in the North Sea. Diving is still an important part of oil production; it is a challenge that we do not fully understand the mechanisms involved. We do not know enough to prevent possible long-term effects of this activity. However, it is important to note that the mechanisms involved in the physiological response to diving could also be involved in its prevention. This makes diving an important tool for studying a number of important disease processes.

Together with Steve Thom and John Ross, I had the opportunity to write the chapter on the physiology and pathophysiology of saturation diving for Comprehensive Physiology that allowed me to focus on this problem [7].

I am now Professor Emeritus in Trondheim and Professor of Environmental Physiology at the University of Aarhus, Denmark.

#### What career choices were you faced with? Why/how did you make the career decisions you did?

I recently found some notes that I made when I started to study medicine. I there wrote that I wanted to avoid boring and routine jobs. I was always looking for new challenges, and my career decisions have been governed by that. I am still looking.

#### What was (were) the main question(s) that you tried to answer in your career? How was this/were these addressed by those in your field and what was your role in the story?

We were some of the first to use ultrasound to study blood flow in the heart and in large vessels. At that time, we met a number of experts on ultrasound who stated that they had the equipment to do this, but they saw no practical use of this. The same argument was used regarding the measuring of bubbles. The clinical use of mathematical models was also thought to be of no practical value.

Physical exercise was claimed to increase the risk of DCS; we were the first to show that physical exercise could significantly reduce bubble formation and by that reduce the risk of injury.

On all accounts, we have, in my opinion, been proven right, showing that Arthur C. Clark was right when he said that “if an old and experienced scientist says that something is possible he is usually right, if he says it is impossible he is usually wrong”. As shown above, even young and inexperienced scientists may often be right!

#### Who were the key people who influenced you?

Professor Jens Glad Balchen and J.S Haldane. Balchen showed me that it was important to have a basic idea and to follow that to the end, regardless of opposition. Haldan was the first environmental physiologist who showed the value of using basic physiology to understand man’s response to his environment [8].

#### Who have you influenced? Who are you proud to have mentored?

All my students. Olav Eftedal is especially worth mentioning; he is presently the Chief Medical Officer of Statoil. Furthermore, Ingrid Eftedal, who is doing work on the genetics of diving.

#### What do you believe to have been your seminal (and/or best) authored paper?

Perhaps the first papers on ultrasound [2], the paper on the circulatory model (JENNY) [9], the paper on exercise and elimination of bubbles [5] and the saturation diving review [7].

#### What are still the major unanswered questions in your field? What ‘truth’ or ‘accepted wisdom’ do you now doubt or think should be questioned?

Diving shows disease mechanisms that are most important because they show that diving can be an important model of pathophysiology with significant and widespread implications. I may have been barking up the wrong tree, by focussing too strongly on the vascular bubbles. I originally thought that, but I may have been wrong, because I now believe that hyperoxia and the production of microbubbles are the main problems and show that diving can be an important model.

#### What is the big unanswered question? How would you answer it?

There are a number of questions related to the use of ultrasound, but this has now developed into a significant field where a large number of researchers are involved. This is not the case in diving that still needs significant research. The effects of oxygen radicals and its defence are perhaps the two unsolved questions that we need to address. An answer to these questions will also open the road to make diving a model of the pathophysiology of a number of severe diseases.

#### What do you still hope to achieve/discover?

The clinical use of models still needs a lot of work, and I would like to make a contribution to that and to decompression that still poses a number of unanswered questions. The effects of hyperoxia and antioxidant defence are still not solved.
